# Long Term Follow-Up of 103 Untreated Adult Patients with Type 1 Gaucher Disease

**DOI:** 10.3390/jcm8101662

**Published:** 2019-10-11

**Authors:** Tama Dinur, Ari Zimran, Michal Becker-Cohen, David Arkadir, Claudia Cozma, Marina Hovakimyan, Sebastian Oppermann, Laura Demuth, Arndt Rolfs, Shoshana Revel-Vilk

**Affiliations:** 1 Gaucher Unit, Shaare Zedek Medical Center, Jerusalem 9103102, Israel; dinurtama@gmail.com (T.D.); azimran@gmail.com (A.Z.); michalbc@szmc.org.il (M.B.-C.); 2 Faculty of Medicine, The Hebrew University of Jerusalem, Ein Kerem, Jerusalem 9112102, Israel; arkadir@gmail.com; 3 Neurological Department, Hadassah-Hebrew University Hospital, Jerusalem 9112002, Israel; 4 Centogene AG, 18055 Rostock, Germany; Claudia.Cozma@centogene.com (C.C.); Marina.Hovakimyan@centogene.com (M.H.); Sebastian.Oppermann@centogene.com (S.O.); Laura.Demuth@centogene.com (L.D.); Arndt.Rolfs@centogene.com (A.R.); 5 Faculty of Medicine, University of Rostock, 18051 Rostock, Germany

**Keywords:** Gaucher disease, type 1, untreated, adults

## Abstract

The introduction of disease-specific therapy for patients with type I Gaucher disease (GD1) was a revolution in the management of patients, but not without cost. Thus, the management of mildly affected patients is still debated. We herein report a long-term follow-up (median (range) of 20 (5–58) years) of 103 GD1 patients who have never received enzymatic or substrate reduction therapy. The median (range) platelet count and hemoglobin levels in last assessment of all but six patients who refused therapy (although recommended and approved) were 152 (56–408) × 10^3^/mL and 13.1 (7.6–16.8) g/dL, respectively. Most patients had mild hepatosplenomegaly. Nine patients were splenectomized. No patient developed clinical bone disease. The median (range) lyso-Gb1 levels at last visit was 108.5 (8.1–711) ng/mL; lowest for patients with R496H/other and highest for patients refusing therapy. This rather large cohort with long follow-up confirms that mildly affected patients may remain stable for many years without GD-specific therapy. The challenge for the future, when newborn screening may detect all patients, is to be able to predict which of the early diagnosed patients is at risk for disease-related complications and therefore for early treatment, and who may remain asymptomatic or minimally affected with no need for disease-specific therapy.

## 1. Introduction

Gaucher disease (GD), one of the most common lysosomal storage disorders, is a multi-system disease known for its great phenotypic heterogeneity [1,2]

The disease is characterized by hepatosplenomegaly, anemia, thrombocytopenia, and skeletal disease. Enzyme replacement therapy (ERT) for patients with type 1 GD (GD1), first introduced in 1991 [3], has proven to be effective in the management of the key disease parameters and preventions of bone-related complications [4]. The enzymatic treatment has also been remarkably safe, with few adverse effects reported [2]. Hence, with predictable efficacy and good safety profile, the major disadvantage of ERT has been the bi-weekly intravenous infusions and very high cost of therapy [5].

Oral substrate reduction therapy (SRT ) has been introduced for the treatment of GD1 in 2000 with miglustat and in 2014 with eliglustat [6,7]. This therapy aims to prevent storage not by correcting the original enzymatic defect but, instead, by decreasing the biosynthesis of the glucocerebroside, resulting in less or no accumulating of the substrate. Although effective, the use of SRT impacts on several metabolic pathways beyond glucocerebroside [8], it has more frequent and severe adverse events than ERT. In the case of eliglustat, there is an added issue of drug-drug interaction due to its dependence on the individual CYP2D6 metabolism and similar cost as all ERTs [5,9]. Quite often, reviews about GD as well as chapters in textbooks fail to emphasize the fact that many patients may remain untreated for many years without any GD-related complications [10].

Herein we report the outcome of adult patients with GD1 who have been followed in our Gaucher Unit for up to six decades. All 103 included patients have never received a single dose of ERT or SRT, highlighting the fact that not every patient with GD needs therapy.

## 2. Methods

**Figure 1:**
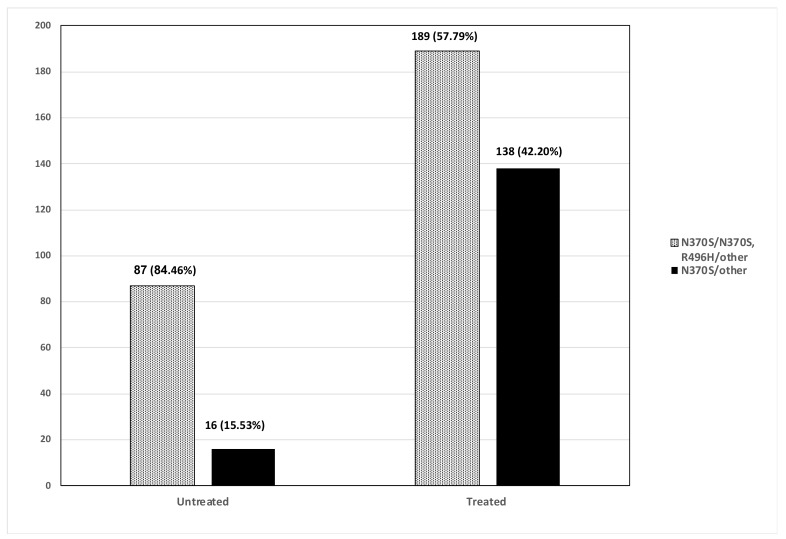
Untreated and treated patients with type I Gaucher disease (≥23 years) who were seen at least once in the Gaucher unit between July 2014–February 2019.

The clinical charts of 440 adult patients (>23 years at time of last evaluation) with GD1 followed in Shaare Zedek Medical Center who were followed for more than five years and evaluated at least once between July 2014 to February 2019 were reviewed (Figure 1).

Patients who have never received any specific treatment (ERT and/or SRT) were included in this analysis. Data from the last clinic visit were collected including medical history, physical examination, laboratory tests and imaging results. Ultrasound assessment of the spleen and liver multiple for normal volume was calculated [11,12,13]. Femoral neck and lumbar spine bone density were measured by dual-energy *X*-ray absorptiometry (DEXA).

Since 2014, glucosylsphingosine (lyso-Gb1) measurement had been included in the routine clinical and laboratory assessment during all follow-up (annual/semiannual) visits. Lyso-GB1 was collected and analyzed from on dry blood spot (DBS) filter cards (CentoCard^®^, Centogene Germany) and sent to Centogene^®^, Rostock, Germany. Lyso-Gb1 levels were measured using mass spectrometry of a sample from dry blood spot, as previously described [14]. The study was approved by SZMC IRB.

### Statistical Analysis

Results are presented as median and range. Kruskal Wallis test with Bonferroni correction for subgroup analysis was used to compare continuous non-normally distributed data between the four study groups. Chi-square test was used to test the association between categorical variables. Correlations between continuous variables were tested by non-parametric Spearman’s correlation. IBM SPSS version 25 was used for analysis. Results were considered to be statistically significant when two-tailed *p*-values were <0.05.

## 3. Results

One hundred and three untreated patients with GD1 followed for a median (range) of 22 (5–58) years were included in the study. Eighty-three (80.5%) patients, from different families and no consanguinity, were found to have the N370S/N370S mutation (Table 1). The clinical characteristics of patients at last follow-up according to the underlying mutation are shown in Table 1. Six patients who refused disease-specific therapy, although recommended and approved, are presented as a separate group. The age of diagnosis was similar for N370S/N370S and compound heterozygous (N370S/other), except for patients with the R496H/other (Table 1). The years of follow-up from diagnosis was similar between groups. Nine patients were splenectomized, of whom eight were splenectomized between 1977 and 1992 (before the ERT era). One male patient had a splenectomy in 2002 prior to referral to our clinic due to splenic artery aneurysm. Four patients developed Parkinson disease during follow-up at the median (range) age of 51.75 (42−60) years. No history of malignancy and monoclonal gammopathy were reported in this cohort.

Thrombocytopenia, i.e., platelet count <100 × 10^3^/mL, was found in 16 patients. Clinically significant thrombocytopenia (<50 × 10^3^/mL) was found only in one patient who refused therapy. All, but one patient, had hemoglobin above 10 g/dL. The one patient with significant anemia was diagnosed with iron deficiency (not related to the GD). The median (range) change of platelet and hemoglobin counts over the years of follow-up were 0 (−84 to 67) and 0.12 (−2.8 to 2.3), respectively; not significantly different between study groups. The median spleen and liver multiple from normal volume was <3 in all groups, except those refusing therapy (Table 1).

Lumbar spine T-scores were available for 71 patients. Signs of osteopenia (T score < −1) and osteoporosis (T score < −2.5) were found in 29 and in 7 patients, respectively. No patient developed clinically significant bone disease during follow-up.

**Figure 2:**
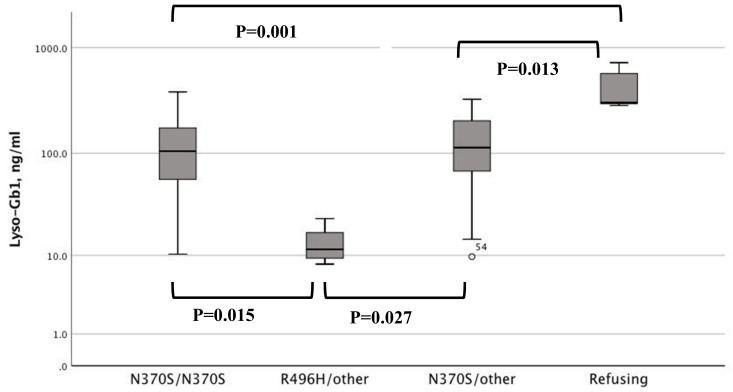
Lyso-Gb1 levels at last assessment in patients with type I Gaucher disease in the different study groups; N370S/N370S, R496H/other, N370S/other and patients refusing therapy.

**Figure 3:**
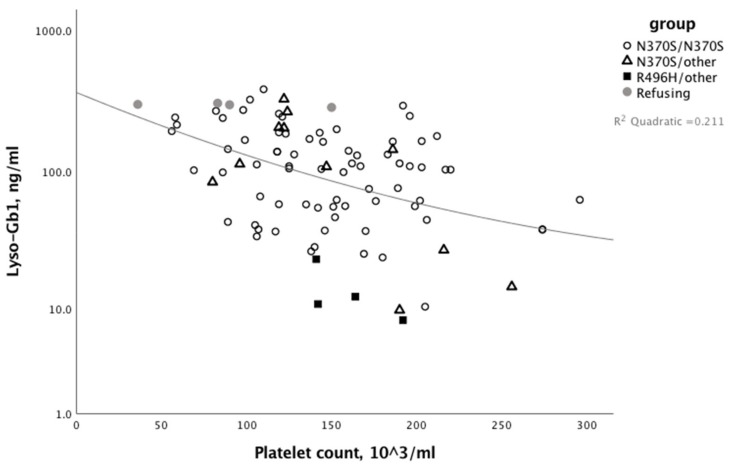
Correlation between platelet count and lyso-Gb1 levels at last assessment in patients with type I Gaucher disease in the different study groups; N370S/N370S, N370S/other, R496H/other, and patients refusing therapy. Splenectomized patients were excluded.

The median (range) lyso-Gb1 levels at last visit was 108.5 (8.1–711) (normal < 8 ng/mL). Patients with R496H/other had the lowest lyso-Gb1 levels and patients refusing therapy had the highest lyso-Gb1 levels (Figure 2). In non-splenectomized patients, the lyso-Gb1 level was negatively correlated with platelet count, but not with any other GD-related parameter (Figure 3).

## 4. Discussion

Enzyme replacement therapy has revolutionized the natural history of many patients with GD worldwide [1]. In parallel, its financial success for the pharmaceutical companies has favorably impacted on the entire field of rare diseases, with the realization that drug development for orphan diseases is profitable, and thereby a growing number of rare diseases now have treatments [15]. On the downside, the significant revenues from each individual patient have created pressure by companies to prescribe higher doses and to widen the indications for therapy [16,17]. The purpose of this report is to provide data that not all patients with GD1 require specific therapy. Obviously, one can translate this lack of treatment to a major saving of hundreds of millions of dollars. This aspect is secondary to the basic issue of the right way to manage minimally affected patients, and it is also based on the old dogma, the so-called Hippocratic injunction, of “primum non nocere” [18].

Admittedly, in the early years of ERT and some countries today, the criteria to administer the costly medication were dictated by restricted funding; however, in Israel, as time moved on, our policy of follow-up of mildly affected patients without specific therapy was truly a reflection of what we believed was good medical practice even before our hypothetical considerations that ERT may increase the likelihood to develop Parkinson disease as a comorbidity [16]. Although many of the experts tend to criticize our approach [19] and we know of many totally asymptomatic patients (mainly in the USA) receiving high dose ERT or, more recently, the new SRT eliglustat, we are not the only center, nor the first one, to follow untreated patients with GD. In 1995, Beutler et al. [19] conducted a retrospective study of 45 patients with GD and concluded that adult patients showed little progression of disease; 15 of his 45 stable patients did not receive ERT; this conclusion was based on blood counts, organ volumes and skeletal lesions, with an average follow-up of over five years (63.7 months) and a median of five years [20]. Fifteen years later, Piran et al. reported on the course of signs and symptoms of 22 Canadian GD patients that have been followed without specific therapy for a median of 9.5 years (range: 3–16 years) [21]. Patients’ hemoglobin, platelet count and spleen volume remained stable over time. Bone disease remained stable over time in most patients, albeit three of those patients developed aging new bone infarcts and one patient (N370S/N370S) developed AVN.

While in the UK some colleagues agreed that not every patient must be treated—for several years children were excluded from this approach, and all were treated with high-dose ERT; this policy has recently been changed (Uma Ramaswami, personal communication). It is for this reason that in the recent review of “How we manage GD in the era of choices” we have delineated two management algorithms—one for adults and one specifically for children with GD—and in both there is an arm for asymptomatic patients (who can be followed indefinitely with no therapy, if there is no disease progression) [2,22]. It should not be a major surprise that every asymptomatic adult used to once be an asymptomatic child with GD.

This is probably the world’s largest series of untreated GD1 patients, and recognition of their long-term (up to few decades) without evidence for disease progression, must be taken into account, whenever there is a consideration of pre-symptomatic “preventive” therapy [2]. Our current observations are going to become more important with the near-future introduction of newborn screening, when the risk of unnecessary therapy may impact on both the actual heath of these children and their psychological development, beyond cost [23,24,25,26].

In this study, we chose to include patients that have been followed in our GD Unit since July 2014 because at that time we added routine testing of lyso-Gb1, currently the most sensitive and specific biomarker for GD [14,27,28]. We selected the minimum age of 23 years in order to have a minimum of 5 years of follow-up of our adult patients. Thirty-three patients started therapy during the study period (2014–2019). None of them developed AVN before or during therapy, ruling out a possible claim that those who developed AVN were taken out of the untreated cohort, thereby introducing a bias.

It is important for the asymptomatic or mildly affected patients to have a periodic (annual or bi-annual) follow-up for GD-related signs and symptoms and development of comorbidities, preferably at a referral center. Splenectomized patients should be further consulted and followed for the risk of developing sepsis, thromboembolic events, and liver disease [29,30,31].

Last but not least, had we chosen to treat all patients with a mild phenotype, based on an estimated average body weight of 70 kg and a low-dose regimen of 30 units/kg/month given for 20 years at a yearly cost of 88,000 dollars per person, we would save hundreds of millions of dollars in our healthcare system, and a much bigger amount for countries using up to four times the dose, and all of that without compromising the patients’ health.

## Figures and Tables

**Table 1:** Characteristic of untreated patients with type I Gaucher disease at last follow-up.

	N370S/N370S	R496H/Other	N370S/Other	Refusing Tx **
**Number**	80	4	13	6
**Female**	49 (60.5%)	1 (25%)	7 (50%)	6 (100%)
Diagnosis age, Y *	22 (0–60)	6 (2–19)	25 (3–40)	13 (5–23)
Last F/U age, Y *	45.5 (22–83)	27 (24–43)	56 (23–72)	46 (22–55)
Time of F/U, Y *	20 (5–58)	22 (5–41)	25 (6–40)	27 (10–46)
PLT, ×10^3^/mL *	163 (56–408)	160 (141–192)	176 (80–364)	103 (36–171)
Hb, ×10^3^/mL *	13 (7.9–16.8)	14.4 (12.9–16)	13.6 (10–16.2)	12.1 (10–14.1)
Spleen, MN *	2.13 (0.51–9.55)	2.39 (2.02–3.41)	2.25 (1.03–9.96)	5.86 (3.57–9.92)
Liver size, MN *	1.07 (0–4.68)	1.22 (1.1–1.63)	1.24 (0.94–5)	3.78 (1.06–8)
T-score lumbar *	−0.75 (−2.9 to 2.8)	−0.5 (−0.8 to −0.2)	−1.4 (−2.5 to 1.3)	−2 (−3.0 to −0.4)
Lyso-Gb1, ng/mL *	104.5 (10.3–381)	11.5 (8.1–23.4)	113 (9.7–325)	301 (284–719)
**Splenectomy**	5	0	2	2
**Parkinson**	2	0	2	0

Tx, treatment; Y, year; F/U, follow-up; PLT, platelet count; Hb, hemoglobin; MN, multiple of normal. * median (range) at last assessment. ** three N370S/N370S, three N370/other. Mutations are described using the traditional amino acid residue numbering, which excludes the first 39 amino acids of the leader sequence.
